# Characterisation of Three Ovine *KRTAP13* Family Genes and Their Association with Wool Traits in Chinese Tan Sheep

**DOI:** 10.3390/ani14192862

**Published:** 2024-10-04

**Authors:** Lingrong Bai, Huitong Zhou, Jianning He, Jinzhong Tao, Jon G. H. Hickford

**Affiliations:** 1 International Wool Research Institute, Faculty of Animal Science and Technology, Gansu Agricultural University, Lanzhou 730070, China; lingrong.bai@lincolnuni.ac.nz (L.B.); huitong.zhou@lincoln.ac.nz (H.Z.); 2 Gene-Marker Laboratory, Faculty of Agriculture and Life Sciences, Lincoln University, Lincoln 7647, New Zealand; 3 College of Animal Science and Technology, Qingdao Agricultural University, Qingdao 266109, China; 4 College of Animal Science and Technology, Ningxia University, Yinchuan 750021, China; tao_jz@nxu.edu.cn

**Keywords:** keratin-associated protein 13 gene (*KRTAP13*), variation, wool traits, fine wool, heterotypic hair fibres, Chinese Tan sheep

## Abstract

**Simple Summary:**

Wool is a fibre with unique physical, chemical, and biological properties, but its quality can be improved with selective breeding to satisfy new needs. To improve our understanding of the genetic mechanisms that underpin variation in fibre traits, this study focused on the characterisation of three genes in the KAP13 family. It revealed that *KRTAP13-2* was associated with the coefficient of variation of fibre diameter and the fibre diameter standard deviation for the heterotypic hair fibres of Chinese Tan sheep. However, *KRTAP13-4* was not associated with any of the wool traits recorded in this study, and *KRTAP13-1* was not variable in the Chinese Tan sheep studied.

**Abstract:**

Understanding the genetic basis of wool traits is crucial for improving wool production. In this study, we investigated the ovine KAP13 gene family, which in humans contains multiple members, while only one member has been identified to date in sheep. Three ovine *KRTAP13* genes, likely representing *KRTAP13-1*, *KRTAP13-2*, and *KRTAP13-4*, were identified through sequence analysis and phylogenetic comparisons. These genes are positioned on chromosome 1, between *KRTAP15-1* and *KRTAP27-1*, in a pattern that is like the arrangement in humans but not identical. Analyses revealed multiple sequence variants of each gene in 356 sheep from a variety of wool, meat, and dual-purpose breeds. The effect of these genes on four fibre traits: mean fibre curvature (MFC), mean fibre diameter (MFD), coefficient of variation of fibre diameter (CVFD), and fibre diameter standard deviation (FDSD), was assessed in 240 lambs of the Chinese Tan sheep breed. An allele of *KRTAP13-2* was revealed to be associated with a decrease in FDSD and CVFD in heterotypic fibres. No associations were found between *KRTAP13-4* variation and wool traits, and an association analysis for *KRTAP13-1* was not conducted because no variation was found in this gene in the Chinese Tan sheep studied. These findings suggest a potential role for *KRTAP13-2* in regulating wool traits, particularly fibre diameter uniformity in larger heterotypic hair fibres, and suggest its potential use as a marker for improving wool traits.

## 1. Introduction

Keratin-associated proteins (KAPs) are components of wool and hair fibres. Typically, a wool or hair fibre comprises the cuticle and cortex, and occasionally they have a medulla, a centrally positioned structure composed of hollow cells within the cortex [1]. Keratin intermediate filaments are embedded in a matrix of the KAPs [2], and consequently the KAPs are thought to have a function in determining the characteristics of wool and hair fibres. 

The KAP proteins are compact in size yet they are abundant in number. Although only 31 KAP genes (designated *KRTAPs*) have been identified in sheep to date [3,4], the human genome contains a larger repertoire, with 89 known *KRTAPs* assigned into 25 KAP families: KAP1 to KAP13, KAP15 to KAP17, and KAP19 to KAP27 [5,6,7]. Among these, the KAP11 and KAP13 families represent the earliest expressed families in human hair follicles, commencing their expression concurrently with the hair keratins and well before the emergence of the first high glycine–tyrosine KAP [5,7,8,9]. The human KAP11 family contains a single member, KAP11-1, while KAP13 comprises four family members.

In human hair follicles, only three KAP13 genes (*KRTAP13-1*, *KRTAP13-2*, and *KRTAP13-3*) demonstrate observable expression, with *KRTAP13-2* displaying strong expression in the cuticle [9]. There is no obvious expression of *KRTAP13-1* in mouse hair follicles, but instead with embryonic mice it is localised in the periderm, the filiform tongue papillae, and the parakeratotic tail epidermis of adult mice [10]. These findings highlight diversity within the KAP13 family and suggest that different family members may serve different functions or exhibit differential behaviours.

To date, only one member of the KAP13 gene family, *KRTAP13-3*, has been described in sheep [11]. The purpose of this study is to search for additional family members and, if found, ascertain whether these genes are polymorphic. This study also aims to investigate whether any variation detected in the genes is associated with fibre traits in Chinese Tan sheep. This indigenous Chinese breed is recognised for growing wool with a distinguishing ‘spring-like’ crimp that is notable up to approximately 35 days of age, a period traditionally referred to as ‘Er-mao’. The pelts of the lambs are important in the fur trade in the colder regions of Northern and Western China. The wool comprises two types of fibres: a ‘fine’ fibre, which is typically non-medulated, and a heterotypic and ‘coarser’ hair fibre that can be medulated.

## 2. Materials and Methods

The collection of sheep blood samples adhered to ethical guidelines outlined in the Animal Welfare Act 1999 (NZ Government) and specifically followed Section 7.5 Animal Identification of the Animal Welfare (Sheep and Beef Cattle) Code of Welfare 2010, which covers the collection of blood drops by nicking sheep ears. 

### 2.1. Sheep Investigated and Wool Trait Measurement

In this study, two groups of sheep were investigated. The first group comprised 116 sheep selected from various farms. They were chosen to be unrelated and to represent breeds that have been selected historically for meat, wool, and dual-purpose production systems. These sheep were chosen to create a diverse base to ascertain the likely extent of DNA sequence variation in the *KRTAP13-n* genes. The sheep comprised 10 breeds from eight unrelated farms and were numbered as follows: South Suffolk (6), Poll Dorset (12), Israeli Assaf (15), Corriedale (10), Romney (20), Texel (10), Merino (20), Coopworth (6), White Dorper (10), and Black Dorper (7). This group was solely used for screening for variation in ovine *KRTAP13-n*. Because no wool samples were collected from these sheep, they were not subjected to gene association analyses.

The second group comprised 246 Chinese Tan lambs. These were the progeny of ten lambs. Most of the lambs were born as singles, but six of them (three pairs) were born as twins. It was decided to remove the twins from the association analyses, as they might confound the results. Consequently, 240 single-born lambs were used in the general linear modeling approach to ascertain if there was a relationship between wool trait variation and variation in the *KRTAP13-n* genes studied.

Wool samples were collected from the mid-side region (above the flank over the 13th rib) of the Tan lambs at Er-mao. For each sample, the larger heterotypic hair fibres and the fine wool fibres were separated based on their difference in fibre diameter and length. To do this, the sample was spread out on a flannel-covered board, and then using an index figure to press the base of all the fibres against the board, the other hand was used to grip the top of the longer heterotypic fibres and pull them out of the fibre sample. The fine wool fibres remained on the flannel-covered board. It was repeated several times to ensure that all the heterotypic hair fibres were separated from the fine wool.

The fine and heterotypic fibres were then measured for mean fibre diameter (MFD), fibre diameter standard deviation (FDSD), coefficient of variation of fibre diameter (CVFD), and mean fibre curvature (MFC). The measurements on the heterotypic fibres were undertaken by the New Zealand Wool Testing Authority, Napier, New Zealand, using IWTO sanctioned methods (IWTO-12-2012), while the measurement of the fine wool samples was undertaken by Pastoral Measurements Limited, Timaru, New Zealand.

A sample of venous blood was collected from each sheep and dotted onto TFN blotting paper (Munktell Filter AB, Falun, Sweden). The DNA in the white blood cells was purified from 1.2 mm punches taken from the dried blood on the TFN paper, using a two-step washing process that was described for ovine *KRTAP19-5* [12]. This method involves incubating the blood card punches in a 20 mM NaOH solution for 30 min at room temperature, aspiration of the NaOH solution, and a subsequent single wash with 1 × TE^−1^ buffer (10 mM Tris–HCl, 0.1 mM EDTA, pH 8.0). The prepared samples were air dried and stored until needed.

### 2.2. Search for KRTAP13-n in the Sheep Genome

The coding sequence of ovine *KRTAP13-3* (JN377429) was used for a BLASTN search of the Sheep Genome Assembly ARS-UI_Ramb_v2.0 (GCA_016772045.1). The genomic sequences that contained open reading frames (ORFs) with high sequence similarity to this coding sequence were presumed to be *KRTAP13-n* family members. The homologous genomic sequences identified were then used to design three sets of PCR primers (Table 1) for amplifying the three separate *KRTAP13-n* identified from the sheep genomic DNA purified as described above.

### 2.3. PCR Amplification and Single Strand Conformation Polymorphism Analysis of KRTAP13-n

PCR primers were synthesised by Integrated DNA Technologies (Coralville, IA, USA). Amplification for all three genes was carried out in 15 μL reactions that contained one prepared punch of the TFN paper, 150 μM of each dNTP (Bioline, London, UK), 0.25 μM of each primer, 2.5 mM Mg^2+^, 0.5 U of Taq DNA polymerase (Qiagen, Hilden, Germany), and 1× the reaction buffer supplied with the enzyme. The thermal profile for amplification of the three genes studied consisted of an initial denaturation step at 94 °C for 2 min, followed by 35 cycles of 30 s at 94 °C, 30 s at the annealing temperature shown in Table 1, and 30 s at 72 °C, and a final extension step for 5 min at 72 °C. The thermal cycling was undertaken in S1000 PCR machines (Bio-Rad, Hercules, CA, USA).

The PCR amplicons were analysed using a single strand conformation polymorphism (SSCP) approach. For each amplicon, a 0.7-μL aliquot of each was mixed with 7 μL of gel loading dye (0.025% bromophenol blue, 0.025% xylene–cyanol, 98% formamide, 10 mM EDTA). After denaturation at 95 °C for 5 min, the DNA samples were cooled on wet ice and loaded on 16 cm × 18 cm, 14% acrylamide/bisacrylamide (37.5:1) (Bio-Rad) gels. Electrophoresis was carried out using the conditions described in Table 1 and using 0.5× TBE buffer in Protean II xi cells (Bio-Rad).

Upon completion of the electrophoretic separation, the SSCP gels were fixed and stained for 10 min in a solution containing 10% ethanol, 0.5% acetic acid, and 0.2% silver nitrate. Next, the gels were rinsed with distilled water and the banding patterns were revealed by developing the gels in a solution of 3% NaOH and 0.1% HCOH. Development was undertaken until dark-staining bands appeared on the yellow background of the gel. At that time, gel development was halted by removing the developer solution and by the addition of a 10% ethanol and 0.5% acetic acid aqueous solution.

### 2.4. DNA Sequencing and Sequence Analysis

The amplicons that were selected for sequencing were identified using the PCR–SSCP approach described above. Specifically, amplicons representing different SSCP banding patterns from sheep that appeared to be homozygous for the amplicon sequence were sequenced three times in both directions at the Lincoln University DNA sequencing facility (Lincoln University, Lincoln, New Zealand) using a Sanger dideoxy approach.

For rare alleles at all three loci, which were exclusively identified in heterozygous sheep, a previously described alternative approach was employed. This was described previously [13]. In this approach, a gel slice corresponding to a SSCP band of the rarer allele is excised from the SSCP gel, macerated, and then used as a template for re-amplification with the original primers. This effectively enables allele-specific amplification, and the resulting second amplicon can be matched to the banding patterns observed on the SSCP gels under the original conditions and then sequenced directly. 

Once the sequencing outputs from both directions of read were obtained in triplicate, they were aligned, translated, and subjected to phylogenetic analysis using DNAMAN XL (version 10, Lynnon BioSoft, Vaudreuil, QC, Canada).

### 2.5. Statistical Analyses

Genetic heterogeneity and polymorphism information content (PIC) were calculated using an online calculator (https://www.genecalculators.net/pq-chwe-polypicker.html; accessed on 17 July 2024). Association analyses were performed using Minitab version 16 (Minitab Inc., PA, USA). General Linear Models (GLMs) were used to assess the effect of the absence or presence of the *KRTAP13-2* and the *KRTAP13-4* alleles on the various wool traits that were measured. The models incorporated sire and lamb sex because sire was identified to have an influence on all the wool traits, while sex was identified as a factor impacting certain wool traits. The model employed was: Y_jkl_ = µ + V_j_ + G_k_ + S_l_ + e_jkl_; where Y_jkl_ is the phenotypic value of the trait, µ is the group raw mean for that particular trait, V_j_ is the effect of the *j*th allele (presence or absence), G_k_ is the effect of lamb sex, S_l_ is the effect of the *l*th sire (10 sires in total), and e_jkl_ is the random residual effect.

## 3. Results

### 3.1. Identification of KRTAP13-n in the Sheep Genome

A BLAST search of the ovine *KRTAP13-3* coding sequence (JN377429) revealed four homologous ORFs in the sheep genome sequence NC_056054.1. These were: g.126091672_126092142 (forward strand, identity = 99%, E value = 0.0), g.126131729_12613232 (reverse strand, identity = 88%, E value = 2 × 10^−143^), g.126086934_126087437 (reverse strand, identity = 85%, E value = 3 × 10^−116^), and g.126079963_126080457 (forward strand, identity = 87%, E value = 1 × 10^−89^). The ORF of g.126091672_126092142 was presumed to be *KRTAP13-3*, as it displayed only one nucleotide difference to the *KRTAP13-3* sequence JN377429. The other three ORFs are likely to be the remaining KAP13 family members and possibly represent the orthologs of human *KRTAP13-1*, *KRTAP13-2*, and *KRTAP13-4*.

**Figure 1:**
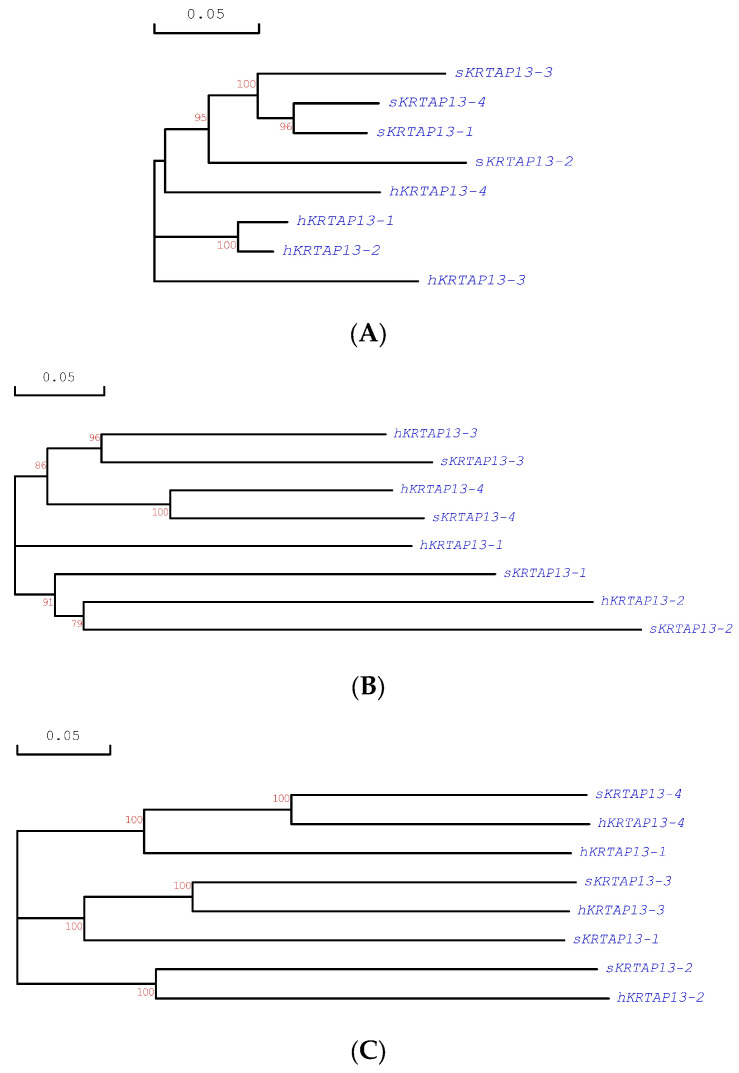
Phylogenetic trees of the human and ovine *KRTAP13-n*. The trees are constructed for the entire coding region (**A**), a 1 kb upstream flanking region (**B**), and a 1 kb downstream flanking region (**C**). Bootstrap confidence values are represented by the numbers at the forks of the trees, and only those equal to or higher than 70% are shown. The scale bar indicates a rate of 0.05 nucleotide substitution per site. The sheep and human sequences that were compared are labeled with the prefixes “s” and “h”, respectively.

To confirm which ORFs correspond to human *KRTAP13-1*, *KRTAP13-2*, and *KRTAP13-4*, phylogenetic analysis was conducted on the sequences of these ORFs, along with the human *KRTAP13-1* to *KRTAP13-4* sequences. However, the coding sequence of the *KRTAP13-n* from one species formed distinct clusters with the other yet was separate from their orthologs in the other species (Figure 1A). Subsequent phylogenetic analyses on the flanking sequences, both upstream and downstream, revealed clustering of orthologs for all the family members except *KRTAP13-1* (Figure 1B,C). Based on these analyses, the ORFs g.126131729_12613232, g.126086934_126087437, and g.126079963_126080457 were assigned the names *KRTAP13-1*, *KRTAP13-4*, and *KRTAP13-2*, respectively.

**Figure 2:**
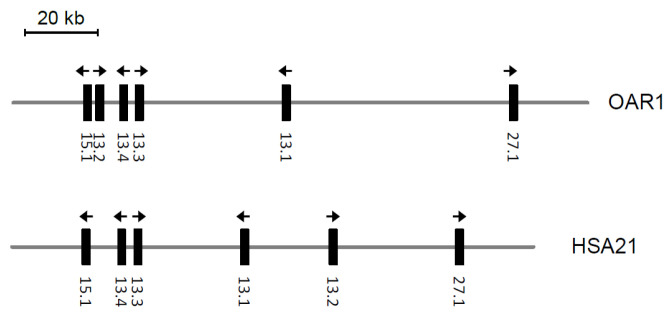
Chromosomal location of *KRTAP13-n* in sheep and humans, together with two flanking *KRTAPs* (*KRTAP15-1* and *KRTAP27-1*). The sheep orthologs are located on chromosome 1 (OAR1), whereas the human orthologs are on chromosome 21 (HSA21). The vertical bars indicate *KRTAPs* with abbreviated names (e.g., 15.1 represents *KRTAP15-1*) being labeled below. The arrows indicate the direction of transcription. The sheep sequence is based on the sheep chromosome 1 genome assembly sequence NC_056054.1 (the forward strand), whereas the human sequence is based on the human chromosome 21 genome assembly NC_000021.9 (the reverse strand).

The ovine KAP13 genes are clustered and located between *KRTAP15-1* and *KRTAP27-1* on chromosome 1, a genetic arrangement similar to that observed in humans (Figure 2). However, the configuration of the KAP13 gene members differs slightly between these two species. In humans, *KRTAP13-2* is positioned between *KRTAP13-1* and *KRTAP27-1*, while in sheep, it is located between *KRTAP15-1* and *KRTAP13-4* (Figure 2).

### 3.2. Variation in Ovine KRTAP13-n

**Figure 3:**
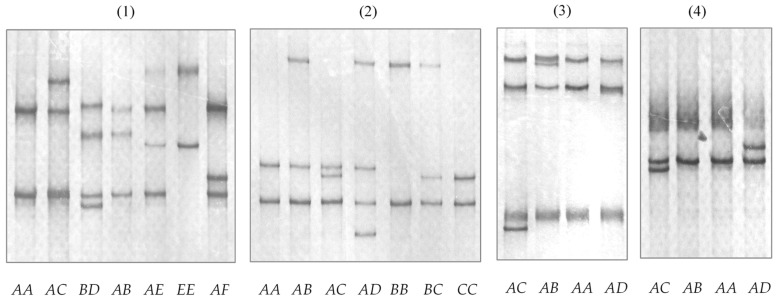
PCR-SSCP patterns of ovine *KRTAP13-n*. (**1**) Six different banding patterns (from *A* to *F*) are observed for ovine *KRTAP13-1*; (**2**) four different banding patterns (from *A* to *D*) are observed for ovine *KRTAP13-2*; and (**3**) and (**4**) four different banding patterns (from *A* to *D*) are resolved for ovine *KRTAP13-4*, but these can only be resolved using two different electrophoresis conditions.

**Figure 4:**
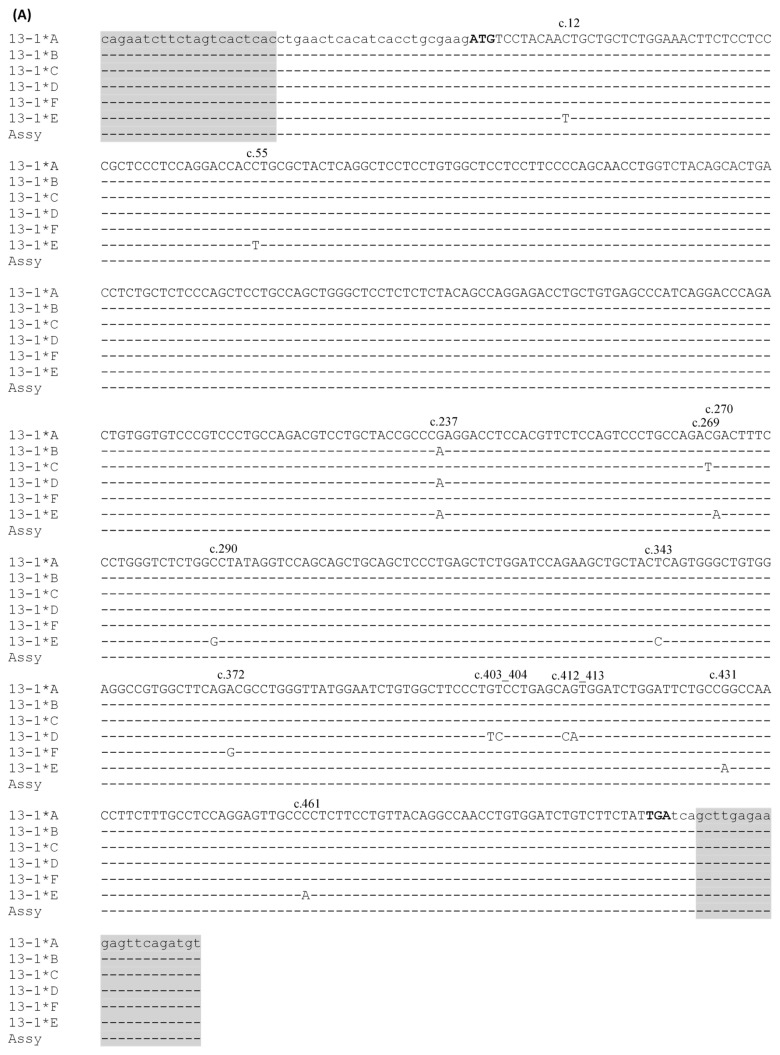

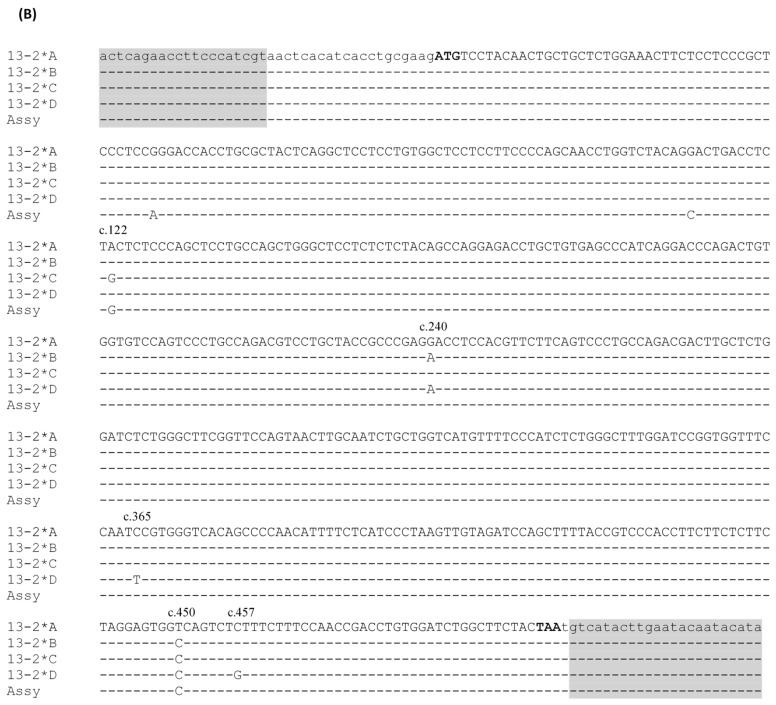

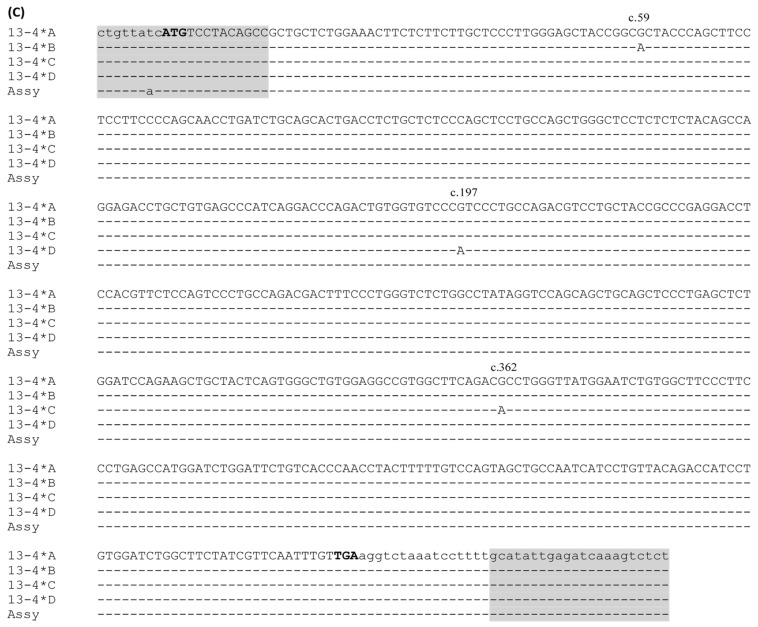
Sequence alignments of ovine *KRTAP13-n*. Allele sequences of *KRTAP13-1* (**A**), *KRTAP13-2* (**B**), and *KRTAP13-4* (**C**) are aligned with the assembly sequence NC_056054.1 (abbreviated as Assy). The allele sequences are denoted with shortened gene names followed by a star and the allele name (e.g., allele *A* of ovine *KRTAP13-1* is labeled as 13-1*A). The nucleotides from the coding regions of the three genes are displayed in uppercase letters, whereas those outside the coding region are presented in lowercase letters. The translation start and stop codons are indicated in bold text. Dashes indicate nucleotide sequences identical to those at the top. The nucleotide differences among alleles are indicated by their positions, with numbering following the recommendations of the Human Genome Variation Society [14]. The PCR primer binding regions are shaded. The nucleotide mismatch in the forward primer binding region of *KRTAP13-4* is deliberately introduced to reduce the complementarity between primers.

Six alleles of ovine *KRTAP13-1* were identified using a PCR-SSCP analysis (Figure 3). Sequencing of these alleles revealed 10 single nucleotide polymorphisms (SNPs) (c.12C/T, c.55C/T, c.237G/A, c.269C/T, c.270G/A, c.290A/G, c.343T/C, c.372A/G, c.431G/A, and c.461C/A) and two double nucleotide polymorphisms (DNPs) (c.403_404GT/TC and c.412_413AG/CA) within the coding region (Figure 4A). The SNPs c.269C/T, c.290A/G, c.343T/C, c.431G/A, and c.461C/A would result in amino acid changes p.Thr90Met, p.Ala97Gly, p.Ser115Pro, p.Arg144Gln, and p.Pro154His, respectively. Both DNPs would lead to single amino acid changes, p.Val135Ser and p.Ser138His. Allele *A* was identical to the *KRTAP13-1* genome assembly sequence NC-056054.1.

For ovine *KRTAP13-2*, four PCR-SSCP patterns representing four alleles were observed (Figure 3). Among these alleles, five SNPs were detected: c.122A/G, c.240A/G, c.365C/T, c.450T/C, and c.457C/G (Figure 4B). Three of these SNPs were non-synonymous and would result in amino acid change: p.Tyr41Cys, p.Ser122Phe, and p.Leu153Val. While all these alleles were highly homologous, none was identical to the *KRTAP13-2* genome assembly sequence NC_056054.1. The nucleotide differences between the alleles and the assembly sequence suggest more allele sequences of the gene may exist.

Ovine *KRTAP13-4* also exhibited four alleles, detected by PCR-SSCP under two different electrophoresis conditions (Figure 3). Three SNPs were found, and all of these were non-synonymous: c.59G/A (p.Arg20His), c.197G/A (p.Arg66His), and c.362G/A (p.Arg121His) (Figure 4C). Allele *A* had a sequence identical to the *KRTAP13-4* sequence in the sheep genome assembly NC_056054.1.

In the 116 sheep used for variation screening, the allele frequencies and genetic diversity information for *KRTAP13-1*, *KRTAP13-2*, and *KRTAP13-4* are presented in Table 2. Among these newly identified *KRTAP13-n* sequences, *KRTAP13-1* had a lower level of heterogeneity and PIC, and *KRTAP13-2* and *KRTAP13-4* exhibited higher levels of variation.

### 3.3. Associations between Variation in KRTAP13-n and Wool Traits

While multiple alleles were detected for *KRTAP13-1* in the mixed pool of breeds, only one allele (allele *A*) was present in the Chinese Tan sheep (Table 2). Accordingly, an association analysis was not carried out for this gene.

Analysis of *KRTAP13-2* revealed the presence of alleles *A*, *B*, and *C* in the Tan sheep group, while allele *D* was not detected (Table 2). Among the four MFD-related traits investigated, associations were detected for FDSD and CVFD, but these were only observed for the heterotypic hair fibres and not the finer wool fibres (Table 3). In this respect, the fine wool had an average phenotypic FDSD of 4.0 ± 0.06 µm and CVFD of 24.3 ± 0.30%, whereas the heterotypic hair fibres had an average FDSD of 8.5 ± 0.09 µm and CVFD of 28.4 ± 0.25% (all values represent mean ± SE). The presence of *B* was associated with a reduction in FDSD and CVFD for heterotypic fibres, while *A* was trending towards increased FDSD and CVFD for the heterotypic fibres.

All alleles of *KRTAP13-4* were detected in the Tan sheep group. However, two alleles were found at a frequency of less than 5% (Table 2), leading to the exclusion of sheep carrying these two rare alleles from the association study. No associations were detected between the variation in *KRTAP13-4* and any of the four fibre diameter-related traits for either the fine wool or heterotypic hair fibres (Supplementary Table S1).

## 4. Discussion

This study identifies three new *KRTAPs* in sheep. The high sequence homology between these *KRTAPs* and ovine *KRTAP13-3*, their similar chromosomal location, and the close phylogenetic relatedness to the human *KRTAP13-n* genes suggest that these new *KRTAPs* represent the other three members of the KAP13 gene family, *KRTAP13-1*, *KRTAP13-2*, and *KRTAP13-4*. With the identification of these genes, the number of *KRTAPs* identified in sheep increases from 31 [3,4] to 34, which possibly only represents one-third of the total number of *KRTAPs* present in this species when compared to humans.

An interesting finding is that the *KRTAP13-n* genes were more closely related to each other within species than to their orthologs from another species for the coding region, but this relationship changed in the immediate flanking regions where the orthologs from the sheep and humans were more closely related. This suggests that the coding and flanking regions of *KRTAP13-n* are subject to distinct selective pressures and/or have evolved through different mechanisms. The coding region appears to have undergone concerted evolution, while the flanking regions show signs of divergent evolution. This kind of evolutionary pattern has been previously reported for the KAP1 family [15], and it may lead to sequence and structure homogeneity within the *KAP13-n* proteins while allowing for regulatory diversity among individual family members.

While the KAP13 family in sheep shares similarities with humans in terms of family members and chromosomal locations, differences exist between these two species. A notable difference is the position of *KRTAP13-2*. It is located at one end of the *KRTAP13-n* cluster in sheep, but it is situated at the other end of the *KRTAP13-n* cluster in humans (Figure 2). Research in sheep suggests a prevalence of long intergenic non-coding RNA (lincRNA) genes near these *KRTAP13-n* genes [3], and in humans, lincRNAs are known to contain a higher proportion of transposable element-derived sequences [16]. It is unknown whether these lincRNAs might have had a role in the movement of *KRTAP13-2* in the chromosome.

The association of *KRTAP13-2* with FDSD and CVFD but not with MFD suggests that variation in this gene primarily influences fibre diameter uniformity and not the average fibre diameter for the sample. That is, regardless of the average fibre diameter, fibre diameter variation measured as either a standard deviation of the mean or as CVFD (=FDSD/MFD × 100) is associated with variation in *KRTAP13-2*. The mechanism underpinning this association remains unclear, but one proposition might be that *KRTAP13-2* affects wool fibre ellipticity. Wool fibres typically exhibit an elliptical rather than circular cross-sectional shape, and it has been suggested that increased FDSD and CVFD are correlated with increased ellipticity [17]. In goats, the ellipticity of cashmere fibres has been linked to cuticle thickness, with thicker cuticles associated with greater fibre ellipticity [18].

In humans, although *KRTAP13-2* expression is detected in the cortex, it exhibits stronger expression in the cuticle [9]. If the expression pattern of the *KRTAP13-2* ortholog in sheep mirrors this, strong cuticular expression would suggest a potential role in regulating cuticle thickness or rigidity. This could, in turn, influence wool fibre ellipticity and subsequently impact the FDSD and CVFD.

Coarse wool fibres, with their larger surface area, are inherently more susceptible to deformation from external forces compared to fine wool fibres. This is consistent with the wool trait data obtained in this study, where the fine wool had lower FDSD and CVFD than the heterotypic hair fibres. The impact of cuticle thickness or rigidity on ellipticity is expected to be less pronounced in fine wool than in coarse wool, which may explain why the effect was only detected for the heterotypic hair fibres and not for the fine wool.

Ovine *KRTAP13-2* is in proximity to *KRTAP15-1* on sheep chromosome 1, with approximately 3.3 kb between them. Variation in *KRTAP15-1* has previously been reported to affect wool yield, but it is also associated with variation in FDSD in Merino × Southdown-cross sheep [19]. This is notable because in this study with Chinese Tan sheep, *KRTAP13-2* was found to be associated with variation in FDSD and CVFD. As these studies used sheep from different breeds and farmed in different environments, caution is however required when comparing the results of the two studies.

In ovine *KRTAP13-2*, allele *B* differs from allele *A* at SNPs c.240A/G and c450T/C, and from allele *C* at SNPs c.122A/G and c.240A/G. The detection of wool trait associations with allele *B* suggests that some of these three SNPs may have a functional effect. However, as alleles *A* and *C* differ in two of these SNPs (i.e., c.122A/G and c.450T/C), the lack of association detected for alleles *A* and *C* suggests the functional effect, if any, is possibly because of SNP c.240A/G. This SNP is synonymous and would not change the protein sequence. However, research has suggested that synonymous SNPs can affect mRNA stability, regulate gene expression, and/or alter protein structures [20,21]. Alternatively, this SNP may be linked to variation in other regions of the *KRTAP13-2* gene or nearby genes that have a functional effect. Further investigation into the upstream and downstream regions of this gene or other nearby genes is therefore required. Regardless, the variation reported here for *KRTAP13-2* appears to have the potential to be developed as a gene-marker to improve wool fibre uniformity.

The lack of association detected between *KRTAP13-4* variation and wool traits in the Tan sheep may be due to the imbalanced frequencies of the alleles. Although two alleles (*A* and *D*) had frequencies above 5% and were included in the association study, allele *A* was very common at 86.9%, while allele *D* occurred at a frequency of 8.4%. This imbalance may have resulted in an insufficient number of animals carrying allele *D* in the small population group studied, reducing the likelihood of associations being detectable. Alternatively, variation in this gene may be associated with other wool traits that were not measured in this study, a possibility that cannot be ruled out.

Interestingly, while *KRTAP13-2* and *KRTAP13-4* are closely placed on the sheep chromosome, they appear to exhibit differences in both the amount of genetic variation and their possible effect on wool traits. This would suggest the mechanisms underlying the variation and the associations detected here require further investigation.

An interesting phenomenon observed in this study is the difference in diversity levels among the members of the KAP13 family. While *KRTAP13-2* and *KRTAP13-4* exhibit similar levels of diversity, *KRTAP13-1* appeared to be conserved in the Tan sheep but polymorphic in the population of sheep of diverse breeds. This raises questions about the factors that influence genetic diversity within the KAP13 family.

Considering that the sheep used for variation screening were randomly selected from diverse breeds farmed for different purposes, it would seem less likely that selective breeding has influenced the diversity observed. Natural selection would also seem unlikely to be a significant factor if the protein encoded primarily functions as a structural component among many others in the wool and hair fibres. However, it is plausible that *KRTAP13-1* is expressed in other tissues or involved in functions related to fitness or survival, despite such functions having not been reported yet. In this respect, in mice, *KRTAP13-1* does not appear to be expressed in hair follicles, but it is found in the periderm of embryonic mice and in the filiform tongue papillae and parakeratotic tail epidermis of adult mice [10]. This would suggest that further investigation into the expression patterns of the *KRTAP13-n* genes, and especially the roles of *KRTAP13-1* in sheep, is warranted.

The allele sequences of ovine *KRTAP13-4* required two different electrophoresis conditions for resolution. In this respect, we explored a range of electrophoresis temperatures (from 4 °C to 35 °C), voltages (from 200 V to 400 V), gel concentrations (from 8% to 12%), and the addition of glycerol to gels. Despite these efforts, no single condition could resolve all the allele sequences of ovine *KRTAP13-4*. While optimisation of electrophoresis conditions typically resolves all gene alleles with one set of conditions, the inability to do so with a single gel system has been reported previously for the porcine leptin gene [22]. This highlights a potential limitation in the detection power of PCR-SSCP analyses for resolving all sequence variation in the genes.

## 5. Conclusions

Among the three genes of the KAP13 family, *KRTAP13-2* was associated with FDSD and CVFD in Tan sheep but not with MFD, which may affect fibre uniformity. However, *KRTAP13-1* had no polymorphism in Tan sheep, and *KRTAP13-4* showed no association with the measured traits. All these genes need further investigation.

## Figures and Tables

**Table 1:** PCR primers and PCR-SSCP electrophoresis conditions used for ovine *KRTAP13-n.*

Gene	Primer Sequence (5′-3′)	Expected Size (bp)	Annealing Temperature (°C)	SSCP Condition
* KRTAP13-1 *	CAGAATCTTCTAGTCACTCAC	572	60	10% gel; at 20 °C and 300 V
	ACATCTGAACTCTTCTCAAGC			
* KRTAP13-2 *	ACTCAGAACCTTCCCATCGT	559	57	10% gel; at 20 °C and 300 V
	TATGTATTGTATTCAAGTATGAC			
* KRTAP13-4 *	CTGTTATCATGTCCTACAGCC	550	58	9% gel; at 7 °C and 390 V, or 26 °C and 180 V
	AGAGACTTTGATCTCAATATGC			

**Table 2:** Allele frequency and genetic diversity information for ovine *KRTAP13-n.*

Gene	Sheep Group	Allele Frequency (%)						Het (%)	PIC (%)
		* A *	* B *	* C *	* D *	* E *	* F *		
*KRTAP13-1*	Mixed pool of breeds (*n* = 116)	82.8	2.1	2.1	1.3	10.8	0.9	30.2	28.4
	Tan sheep (*n* = 240)	100.0	-	-	-	-	-	0	0
*KRTAP13-2*	Mixed pool of breeds	45.8	15.0	36.7	2.5	-	-	63.2	56.0
	Tan sheep	64.4	22.9	12.7	-	-	-	51.2	45.8
*KRTAP13-4*	Mixed pool of breeds	50.0	25.9	3.9	20.2	-	-	64.1	58.0
	Tan sheep	86.9	2.5	2.2	8.4	-	-	23.7	22.4

Het: heterozygosity; PIC: polymorphism information content.

**Table 3:** Association of *KRTAP13-2* alleles with wool traits in heterotypic hair fibres of Chinese Tan sheep.

Trait ^1^	Allele Analysed ^2^	Other Alleles Fitted	Mean ± SE ^3^		*p* Value
			Absent	Present	
MFD (µm)	* A * * B * * C *	None None None	29.6 ± 0.62 29.6 ± 0.39 29.8 ± 0.35	29.7 ± 0.34 29.8 ± 0.41 29.2 ± 0.53	0.858 0.680 0.210
FDSD (µm)	* A * * B * * C *	None None None	*7.9 ± 0.28* **8.6 ± 0.18** 8.3 ± 0.16	*8.3 ± 0.15* **8.0 ± 0.18** 8.3 ± 0.24	*0.068* **0.008** 0.936
	* A * * B *	* B * * A *	8.0 ± 0.29 **8.4 ± 0.23**	8.3 ± 0.15 **7.9 ± 0.19**	0.395 **0.037**
CVFD (%)	* A * * B * * C *	None None None	*26.6 ± 0.82* **28.7 ± 0.51** 27.8 ± 0.47	*28.1 ± 0.46* **27.0 ± 0.54** 28.4 ± 0.71	*0.053* **0.002** 0.361
	* A * * B *	* B * * A *	27.3 ± 0.86 **28.4 ± 0.68**	27.9 ± 0.46 **26.8 ± 0.57**	0.429 **0.012**
MFC (°/mm)	* A * * B * * C *	None None None	46.4 ± 1.31 46.4 ± 0.84 46.6 ± 0.74	46.5 ± 0.73 46.7 ± 0.87 45.9 ± 1.12	0.902 0.707 0.478

^1^ MFD—mean fibre diameter; FDSD—fibre diameter standard deviation; CVFD—coefficient of variation of fibre diameter; MFC—mean fibre curvature. ^2^ Among the 240 Tan sheep, allele *A* was absent in 33 individuals and present in 207 individuals; allele *B* was absent in 148 individuals and present in 92 individuals; allele *C* was absent in 184 individuals and present in 56 individuals. ^3^ Predicted means and standard errors derived from GLMs. Values with *p* < 0.05 are shown in bold, while those with *p* < 0.10 are italicized.

## Data Availability

The raw data supporting the conclusions of this article will be made available by the authors on request.

## Supplementary Materials

The following supporting information can be downloaded at: https://www.mdpi.com/article/10.3390/ani14192862/s1. Table S1: Association of common *KRTAP13-4* genotypes with four fibre diameter related measurements from Chinese Tan sheep.
