# Supplementary material for: Characterisation of Three Ovine KRTAP13 Family Genes and Their Association with Wool Traits in Chinese Tan Sheep
Source: Animals (Basel). 2024 Oct 4;14(19):2862. doi: 10.3390/ani14192862 (PMC11476196; doi:10.3390/ani14192862)
Supplement: Supplementary file 1 [file animals-14-02862-s001.zip › animals-3174829-supplementary.pdf]

Supplementary Table S1. Association of common *KRTAP13-4* genotypes with four fibre diameter related measurements from Chinese Tan sheep

**Fibre type**	**Fibre trait**[1]	**Mean ± SE**[2]		***P* value**
		AA (n = 190)	*AD* (n = 39)	
**Fine wool**	MFD (μm)	16.7 ± 0.18	16.9 ± 0.30	0.375
	FDSD (μm)	4.2 ± 0.13	4.3 ± 0.21	0.713
	CVFD (%)	25.0 ± 0.60	24.9 ± 1.02	0.962
	MFC (°/mm)	63.6 ± 1.18	63.0 ± 2.00	0.719
**Heterotypic hairs**	MFD (μm)	29.7 ± 0.36	29.4 ± 0.62	0.649
	FDSD (μm)	8.3 ± 0.16	8.2 ± 0.28	0.874
	CVFD (%)	27.8 ± 0.48	27.9 ± 0.83	0.891
	MFC (°/mm)	46.7 ± 0.77	46.8 ± 1.33	0.988

[1] MFD – mean fibre diameter; FDSD – fibre diameter standard deviation; CVFD – coefficient of variation of fibre diameter; MFC – mean fibre curvature. [2] Predicted means and standard errors of those means derived from GLMMs.
